# Understand the effect of genetic background on the molecular heterogeneity of brain aging

**DOI:** 10.1002/alz70855_101659

**Published:** 2025-12-23

**Authors:** Yi Juin Liew, Michael Maclean, Gareth R Howell, Gregory W Carter, Asli Uyar

**Affiliations:** ^1^ The Jackson Laboratory, Bar Harbor, ME, USA

## Abstract

**Background:**

Age is a major risk factor for Alzheimer's disease (AD), with brain aging heterogeneity influencing individual susceptibility. Studying variability in aging trajectories due to genetic background can help distinguish normal brain aging from early pathological processes. We have previously showed that the profound impact of genetic variation in modulating phenotypes associated with Alzheimer's disease (AD). In this study, we aim to identify normal brain aging signatures across nine wild‐type strains to capture biological variability often overlooked in single‐strain studies, such as those limited to C57BL/6J.

**Method:**

Brain hemispheres from female and male mice (*n* = 4 per sex) across three age timepoints (4, 12, and 24 months) from nine wild‐type strains (A/J, C57BL/6J, 129S1/SvImJ, NOD/ShiLtJ, NZO/HlLtJ, CAST/EiJ, PWK/PhJ, WSB/EiJ and BALB/cJ) were processed for RNA‐Seq. Strain‐specific aging signatures were identified by comparing 12‐ and 24‐month data to the 4‐month timepoint. Differential expression (DE) and gene set enrichment analyses identified gene modules and pathways associated with brain aging. WGCNA was used to identify common aging signatures across strains. Strain‐specific signatures were aligned with human AD‐related modules using AD biodomains to examine brain aging in the context of AD.

**Result:**

At 12 months, DE genes and enriched pathways were limited and highly variable across strains compared to 4 months. By 24 months, distinct strain‐specific aging signatures emerged, alongside common disruptions in immune response and inflammation. Notably, the magnitude and rate of these disruptions varied across strains, suggesting that baseline genetic differences play a critical role in shaping aging trajectories. Aligning these signatures with Alzheimer's disease (AD) biodomains revealed strain‐specific overlaps with AD‐related processes: NOD mice showed synaptic downregulation, WSB, NZO, and 129 strains exhibited proteostasis disruptions, and CAST mice displayed elevated lipid metabolism.

**Conclusion:**

These findings reveal both shared and strain‐specific molecular mechanisms of brain aging, emphasizing genetic influences on neurodegeneration risk. Aging signatures become more pronounced over time, with significant pathway enrichment emerging at 24 months, suggesting an acceleration of aging‐related molecular processes. Studying heterogeneous aging trajectories is crucial for capturing dynamic changes with age while also highlighting baseline genetic differences that shape these processes.

